# Is self-monitoring an effective option for people receiving long-term vitamin K antagonist therapy? A systematic review and economic evaluation

**DOI:** 10.1136/bmjopen-2015-007758

**Published:** 2015-06-25

**Authors:** Pawana Sharma, Graham Scotland, Moira Cruickshank, Emma Tassie, Cynthia Fraser, Christopher Burton, Bernard Croal, Craig R Ramsay, Miriam Brazzelli

**Affiliations:** 1 Health Services Research Unit, University of Aberdeen, Aberdeen, UK; 2 Health Economics Research Unit, University of Aberdeen, Aberdeen, UK; 3 Division of Applied Health Sciences, University of Aberdeen, Aberdeen, UK; 4 Department of Clinical Biochemistry, University of Aberdeen, Aberdeen, UK

**Keywords:** CARDIOLOGY, HEALTH ECONOMICS

## Abstract

**Objectives:**

To investigate the clinical and cost-effectiveness of self-monitoring of coagulation status in people receiving long-term vitamin K antagonist therapy compared with standard clinic care.

**Design:**

Systematic review of current evidence and economic modelling.

**Data sources:**

Major electronic databases were searched up to May 2013. The economic model parameters were derived from the clinical effectiveness review, routine sources of cost data and advice from clinical experts.

**Study eligibility criteria:**

Randomised controlled trials (RCTs) comparing self-monitoring versus standard clinical care in people with different clinical conditions. Self-monitoring included both self-management (patients conducted the tests and adjusted their treatment according to an algorithm) and self-testing (patients conducted the tests, but received treatment recommendations from a clinician). Various point-of-care coagulometers were considered.

**Results:**

26 RCTs (8763 participants) were included. Both self-management and self-testing were as safe as standard care in terms of major bleeding events (RR 1.08, 95% CI 0.81 to 1.45, p=0.690, and RR 0.99, 95% CI 0.80 to 1.23, p=0.92, respectively). Self-management was associated with fewer thromboembolic events (RR 0.51, 95% CI 0.37 to 0.69, p≤0.001) and with a borderline significant reduction in all-cause mortality (RR 0.68, 95% CI 0.46 to 1.01, p=0.06) than standard care. Self-testing resulted in a modest increase in time in therapeutic range compared with standard care (weighted mean difference, WMD 4.4%, 95% CI 1.71 to 7.18, p=0.02). Total health and social care costs over 10 years were £7324 with standard care and £7326 with self-monitoring (estimated quality adjusted life year, QALY gain was 0.028). Self-monitoring was found to have ∼80% probability of being cost-effective compared with standard care applying a ceiling willingness-to-pay threshold of £20 000 per QALY gained. Within the base case model, applying the pooled relative effect of thromboembolic events, self-management alone was highly cost-effective while self-testing was not.

**Conclusions:**

Self-monitoring appears to be a safe and cost-effective option.

**Trial registration number:**

PROSPERO CRD42013004944.

**Strengths and limitations of this study**

- The study is the most up-to-date evidence synthesis on this topic, with the largest number of included randomised controlled trials.
- Clinical heterogeneity was observed among included trials.
- The majority of the trials included participants with mixed clinical indications for anticoagulation therapy, which made it challenging to extrapolate the results to specific clinical populations.
- The perspective of the economic modelling was that of the National Health Service (NHS) and personal social services, and did not capture any wider benefit.
- Long-term outcomes data on self-management from larger cohorts of people with different clinical indications are needed.

## Introduction

Approximately 2% of the population are prescribed long-term oral anticoagulant drugs for atrial fibrillation (AF),^1–3^ heart valve disease,^4–6^ or other conditions with high risk of thrombosis.^7–9^ Historically, treatment has been with vitamin K antagonist therapy, with dose-adjusted warfarin the most commonly used drug. Recently, new oral anticoagulants (NOACs) which do not require dose adjustment, such as dabigatran etexilate, rivaroxaban or apixaban, have been proposed as a possible alternative to warfarin for the treatment of AF.^10^ ^11^ However, NOACs are unsuitable for people with artificial heart valves (AHF), people with liver or renal dysfunctions and those who are taking concurrent medication, which may react with this class of anticoagulants. For these people, warfarin remains the long-term treatment of choice. Furthermore, the lack of long-term evidence on these novel anticoagulants compared with vitamin K antagonists induces some caution in their wide prescription.^10^

Typically, dose adjustment of vitamin K antagonist therapy involves a blood test of clotting (international normalised ratio, INR) with dose titration to maintain this within a narrow therapeutic range (TTR).^12^ ^13^ Underdosing of anticoagulation therapy increases the risk of thromboembolism, while overdosing increases the risk of bleeding events. Repeated and regular measurements of INR, with dose adjustment when necessary, are necessary to ensure safe and effective anticoagulation therapy.^14^

Monitoring of anticoagulant treatment can be delivered in a number of different ways. These include full service provision in specialist anticoagulation clinics, in physician offices or general practices (either with samples sent to a laboratory or with near-patient testing) or self-monitoring^8^ in which patients carry out their own tests at home using approved portable coagulometers, which test a finger-prick blood sample. Self-monitoring includes both self-management, in which patients conduct tests and adjust their treatment according to an algorithm; in self-testing, the patients conduct the tests, but obtain treatment recommendations from a clinician after sending them the results.

Several coagulometers are available, which have CE marketing authorisation and Food and Drug Administration (FDA) approval; these include the CoaguChek system (versions S and XS) (Roche Diagnostics, Basel, Switzerland), the INRatio2 PT/INR monitor, (Alere Inc., San Diego, California, USA) or the ProTime Microcoagulation system (International Technidyne Corporation, ITC—Nexus Dx, Edison, New Jersey, USA). Their precision and accuracy compared with conventional laboratory-based clinical testing have been reported in a number of studies in the literature.^15–17^

The increased use of oral anticoagulants has intensified pressure on healthcare resources.^18^ The use of point-of-care coagulometers for self-monitoring may avoid unnecessary visits to hospitals or clinics while permitting more frequent INR monitoring and timely adjustment of warfarin dosing to avoid adverse events.^19^ The evidence for the effectiveness of self-monitoring is limited^20^ and previously published economic evaluations have produced conflicting results.^14^ ^16^ The aim of this study is to assess the current evidence on the clinical and cost-effectiveness of self-monitoring (self-testing and self-management) in people receiving long-term vitamin K antagonist therapy as an alternative to standard anticoagulation monitoring care. We focus mainly on the current generation of point-of-care devices (eg, CoaguChek XS), which utilised the most recent technology to minimise measurement inaccuracies.

## Methods

### Clinical effectiveness

The methods of the systematic review of clinical effectiveness were prespecified and detailed in a research protocol (http://guidance.nice.org.uk/DT/16/FinalProtocol/pdf/English), and reported according to standard guidelines.^21–24^

#### Identification of studies

We identified a relevant systematic review published in the Cochrane Library in 2010 by Garcia-Alamino *et al*,^20^ which included studies published up to 2007, and had similar objectives to those of this study. Thus, the literature searches for this study were run in May 2013 for the period ‘2007-to date’ to identify newly published reports. All randomised controlled trials (RCTs) included in the Garcia-Alamino *et al*’s^20^ review were obtained and included for full-text assessment. Major electronic databases such as MEDLINE, MEDLINE In-Process & Other Non-Indexed Citations, EMBASE, Biosis, Science Citation Index, and Cochrane Controlled Trials Register (CENTRAL) were searched for relevant primary studies. Evidence syntheses’ reports, conference abstracts (2011–2013), and ongoing studies were sourced from relevant databases. Reference lists of included studies were perused for additional publications and experts in the field contacted for further information on relevant outcomes and ongoing research in the field. Searches were restricted to publications in English. Full details of the search strategies are presented in online supplementary appendix 1.

#### Inclusion and exclusion criteria

We included RCTs comparing self-testing and/or self-management of anticoagulation control using point-of-care coagulometers with standard monitoring care, which consisted of INR monitoring managed by healthcare professionals. We included studies of both adults and children with heart valve disease (eg, AHV), AF or other clinical indications who required long-term vitamin K antagonist therapy. Main outcomes of interest were: (1) major bleeding and thromboembolic events; (2) all-cause mortality; (3) anticoagulation control measured as time and INR values in TTR, and other intermediate outcomes (including frequency of testing, frequency of visits to clinics, patient compliance with testing).

#### Study selection and data extraction

Two authors independently screened the results of the literature searches, retrieved full-text copies of selected studies and extracted relevant data (PS, MC). Information on study design, characteristics of participants, settings, characteristics of interventions and comparators, and outcome measures was recorded for all included studies. The Cochrane Risk of Bias tool was used to assess the risk of bias in the included studies.^22^ Critical assessments of selection, detection, attrition and reporting biases were performed initially by one author (PS) and cross-checked by a second author (MC). Studies were not excluded purely on the basis of their potential risk of bias. Any uncertainty or disagreements during the study selection, data extraction and risk of bias assessment was resolved by discussion or arbitration by a third author (MB).

#### Data analysis

Where appropriate, pooled summary estimates were calculated using Review Manager, software (Review Manager V.5.2, Copenhagen: the Nordic Cochrane Centre, The Cochrane Collaboration, 2012). In the presence of either clinical or statistical heterogeneity, a random effects model was chosen as the preferred method for pooling the effect sizes.^21^ Relative risk (RR) together with 95% CIs were calculated for dichotomous data (Mantel-Haenszel method), while weighted mean difference (WMD) together with 95% CI were calculated for continuous data (inverse-variance method). Where SDs were not given, these were extrapolated, if possible, using test statistics. Heterogeneity across studies was explored by means of the χ^2^ statistic (with significance level at p<0.05) and the extent of inconsistency between studies quantified by means of the I^2^ statistic. For trials that had multiple arms contributing to different subgroups, the control group was subdivided into two groups to avoid a unit of analysis error.

### Cost-effectiveness analysis

A *de novo* Markov model was developed^25^ ^26^ in TreeAge Pro (TreeAge Software, Williamstown, Massachusetts, 2013) to assess the cost-effectiveness of self-monitoring (self-testing and self-management). The model structure was based on previous economic models of INR self-monitoring published in the literature,^14^ ^27–34^ including models assessing the cost-effectiveness of NOAC drugs compared with warfarin in people with AF.^11^ ^35^ In addition, an unpublished economic model was provided by Roche Diagnostics, the manufacturer of the CoaguChek XS coagulometer (J Craig, York Health Economics Consortium, 2013). The model was built and analysed in accordance with the National Institute for Health and Care Excellence (NICE) reference case for the evaluation of diagnostic tests and devices.^36^

#### Model framework and method of synthesis

**Figure 1:**
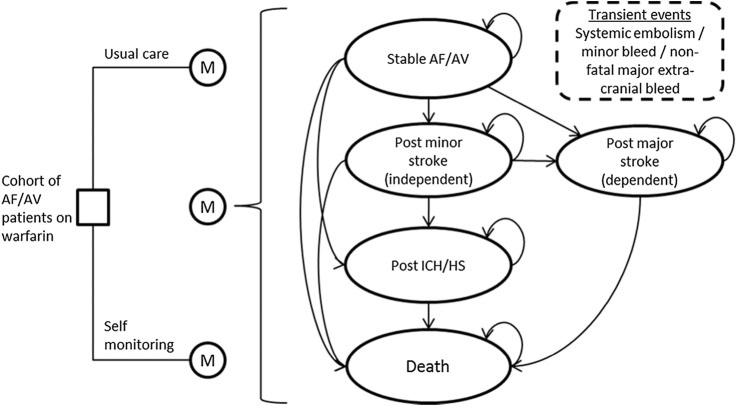
Schematic of the model structure.

The model was populated using data derived from the systematic review of clinical effectiveness, other relevant reviews to inform key parameters (eg, baseline risks), and routine sources of cost data,^37^ ^38^ and information provided by clinical experts. The alternative monitoring pathways were embedded in a Markov model simulating the occurrence of adverse events over time for a hypothetical cohort of people with AF or AHV (figure 1). The model incorporated the pathways of care that individuals currently follow under standard practice in the National Health Services (NHS)—standard monitoring in primary care or in secondary care—as well as proposed pathways for self-testing and self-management. The cost-effectiveness of self-monitoring was assessed as a whole assuming a 50:50 split between self-testing and self-management. The model simulated transitions between the discrete health states on a quarterly (3-month) cycle. Appropriate costs and quality of life weights were attached to modelled events and health states, allowing cumulative health and social care costs and quality adjusted life years (QALYs) to be modelled over time. Full details of the modelling methods are provided in online supplementary appendix 2. The main assumptions made for the base case analysis are summarised in table 1. For the purpose of this study, it was assumed that self-monitoring patients use the CoaguChek XS system.

**Table 1:** Main assumptions made for the base case analysis and justification

Assumptions	Justification
66.45% of standard care monitoring occurs in primary care with practice nurses	Based on previous TAR (manufacturers submission for TA256)^70^
60% of the cohort have atrial fibrillation, 40% have an artificial heart valve	In line with the observed proportions of patients with these conditions in self-monitoring trials^68^
Average age of the cohort is 65 years, and 55% are male	In line with the observed mean age of included patients with these conditions in self-monitoring trials^68^
50% of self-monitoring people self-test, 50% self-manage	Self- assumption
The increase in the number of tests performed per year with self-monitoring is 23	In line with the observed frequency of self-testing in self-monitoring trials^68^
Relative treatment effects are estimated and applied separately for self-testing and self-management	Derived from the observed event rates in cohorts of people being managed under current standard models of care. Relative risks of these events resulting from improved/reduced INR control, conferred by self-monitoring, were derived from the meta-analysis of RCTs of self-monitoring versus standard practice. (see section on clinical effectiveness results)
15% of participants do not commence self-monitoring following training	Based on the RCT literature^43^ and the expert advisory committee consultation
10% of participants discontinue self-monitoring within a year of commencing	Based on consideration of the views of the expert advisory committee (∼5%) and a rate of 14% reported in the largest UK-based trial.^43^
Self-monitoring device costs are annuitized over 5 years to account for the potential for loss and accidental damage	It was assumed that the NHS would pay for devices and loan them out to patients. As such they were annuitized over their expected useful life, to provide an equivalent annual/quarterly cost of use
75% of devices are reused by another patient when a patient discontinues self-monitoring	In line with a previous UK-based economic evaluation^71^

TAR, technology assessment report; INR, international normalised ratio; RCT, randomised control trial; NHS, National Health Service.

The results of the model are presented in terms of a cost-utility analysis (ie, costs for and number of QALYs generated by each monitoring strategy). Self-monitoring strategies were compared to standard care monitoring, to estimate the incremental costs per QALY gained. Both costs and benefits (QALYs) were discounted at a rate of 3.5% per annum, in line with the NICE reference case.^36^ Cost are expressed in 2011/2012 Stirling. The model was initially analysed over a 10-year period, but the impact of adopting longer time horizons was explored through sensitivity analyses. Further sensitivity analyses focused on the standard care comparator (primary care, secondary care), the proportional split between the active interventions (self-testing, self-management), the baseline risk of thromboembolic events and the RRs associated with self-testing and self-management. In addition, cost-minimisation scenarios were considered (assuming an equal number of tests with self-monitoring and standard care, and equivalence in effects). Finally, the results of probabilistic sensitivity analyses were used to express the parameter uncertainty surrounding the base estimates of cost-effectiveness.

## Results

### Clinical effectiveness

Of the 658 records retrieved, 26 RCTs published in 45 papers with a total of 8763 participants met the inclusion criteria. Of the 26 included RCTs, 21 trials with a total of 8394 participants provided suitable data for statistical analyses relevant to the comparisons and outcomes of interest. A flow diagram outlining the selection process is shown in online supplementary appendix 3.

The 26 included trials were conducted in Europe and North America. Seventeen trials (17/26) compared self-management with standard care,^39–55^ six assessed self-testing,^56–61^ and one evaluated both self-testing and self-management versus either trained or untrained routine care (four arms).^62^ The remaining two trials compared self-testing with self-management,^63^ ^64^ one of which focused exclusively on children.^63^ Two trials enrolled exclusively participants with AF,^55^ ^59^ six trials limited inclusion to participants with AHV^41^ ^45^ ^46^ ^50^ ^52^ ^56^ and 18 trials^39^ ^40^ ^42–44^ ^47–49^ ^51^ ^53^ ^54^ ^57^ ^58^ ^60–64^ included participants with mixed clinical indications. The majority of the included trials (22/26) used the CoaguChek system for INR monitoring. Two trials used either INRatio or the CoaguChek S for INR measurement (but did not present results according to the type of the point-of-care device used),^44^ ^56^ while the other two trials used the ProTime system.^53^ ^60^

Table 2 summarises the characteristics of the included trials (full details are shown in online supplementary appendix 4 table S1). The included trials varied in size (16–2922 participants), the length of study duration (3.5–57 months), the age of the included adult participants (16–91 years) and the type of standard care (63.6% of the participants measured INR in secondary care, 27.2% in primary care and 9.2% in mixed care setting). In approximately 95% of the included participants, mean age was between 50 and 70 years. Nine trials, which includes 75% of the total participants, had study duration of more than or equal to 12 months.^41^ ^43^ ^46^ ^47^ ^50–52^ ^60^ ^63^ Three trials recruited participants who were new to anticoagulation therapy,^46^ ^48^ ^51^ two trials included participants receiving anticoagulants for the past 1–2 months,^53^ ^61^ 12 trials recruited participants who had been on anticoagulants for at least 3 months before randomisation^39^ ^40^ ^42^ ^43^ ^47^ ^54^ ^57–59^ ^62–64^ while the remaining trials did not provide this information.

**Table 2:** Summary of the characteristics of included trials

Characteristics	Range	Total number (%)	Number of trials
Sample size, n	16–2922	8763	26
Self-monitoring, n		4553 (51.9)	
PSM	14–579	2619 (57.5)	20*
PST	14–1465	1934 (42.5)	9*
Standard care, n		4199 (47.9)	
AC clinic	17–1457	2669 (63.6)	15
GP/physician	26–576	1143 (27.2)	6
AC clinic or GP/physician	49 to103	387 (9.2)	5
Study duration, months	3.5–57†		
<12	16–320	2186 (25)	17
≥12	28–2922	6577 (75)	9
Age, years	1–91		
Mean age groups, years			
Mean age ≤18	1–19	28 (<1)	1
Mean age >18 to <50	22–71	100 (∼1)	1
Mean age ≥50 to <70	16–91	8289 (94.6)	21
Mean age ≥70	65–91	85 (∼1)	1
Clinical indication, n			
AF	85–202	287 (3)	2
AHV	58–1155	2434 (28)	6
Mixed indication	16–2922	6042 (69)	18
POC devices, n			
CoaguChek	28–1155	5479 (62.5)	22
ProTime	140–2922	3062 (35.0)	2
INRatio2	–	0	0
CoaguChek+INRatio2	16–206	222 (2.5)	2
Outcomes, n			
Thromboembolic events	49–2922	8394 (95.8)	21
Bleeding events	49–2922	8394 (95.8)	21
Mortality	49–2922	6537 (74.6)	13
Time in therapeutic range	28–2922	6245 (71.3)	18
INR values in range	49–1155	4472 (51)	12

*For conversion of study duration reported in week, 4 weeks was considered equivalent to 1 month.

†Three of the 26 trials reported both PSM and PST arms.^62–64^

PSM, patient self-management; PST, patient self-testing; AC, anticoagulation; GP, general practitioner; AF, atrial fibrillation; AHF, artificial heart valves; POC, point-of-care; INR, international normalised ratio.

**Figure 2:**
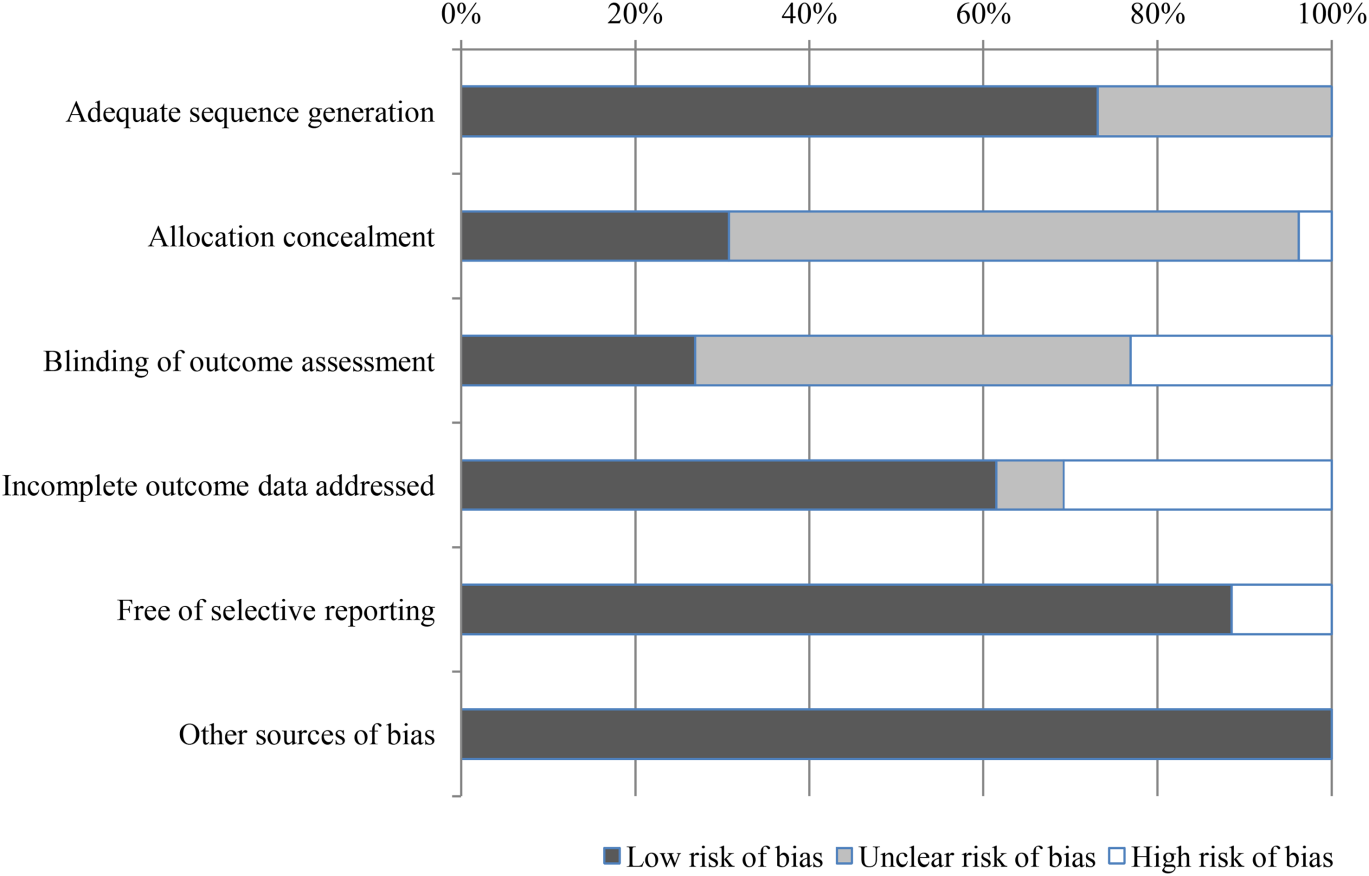
Summary of risk of bias of all included studies.

Only four trials were assessed to have adequate sequence generation, concealed allocation and blinded outcome assessment and therefore were judged at low risk of bias.^47^ ^61^ ^63^ ^65^ The remaining trials were judged at ‘unclear’^40–46^ ^48–50^ ^52^ ^56^ ^58^ ^59^ ^62^ ^64^ or ‘high’^39^ ^53–55^ ^57^ ^60^ risk of bias (figure 2) (full details of the risk of bias assessment are presented in online supplementary appendix 4 table S2).

#### Major clinical outcomes

Major bleeding and major thromboembolic events were reported in the majority of trials. Definitions varied between the trials and not all trials used well-defined criteria. In general, major events (bleeding or thromboembolic) were defined as complications requiring hospital admission or medical assessment. Fatal bleeding and thromboembolic events were counted as deaths.

Table 3 shows the main findings of self-monitoring (self-testing and self-management) compared with standard clinical monitoring.

**Table 3:** Meta-analyses results of major clinical outcomes and time in therapeutic range

Outcomes	Self-monitoring		Standard care		RR (95% CI)	p Value	Number of trials
	Number of events	Total number	Number of events	Total number			
All bleeding	736	4278	736	4116	0.95 (0.74 to 1.21)	0.66	22*
Self-management	250	2403	310	2237	0.94 (0.68 to 1.30)	0.69	15
Self-testing	486	1875	426	1879	1.15 (1.03 to 1.28)	0.02	7
Major bleeding	247	4188	231	4014	1.02 (0.86 to 1.21)	0.82	21*
Self-management	96	2403	78	2237	1.08 (0.81 to 1.45)	0.60	15
Self-testing	151	1785	153	1777	0.99 (0.80 to 1.23)	0.92	6
Minor bleeding	489	2757	505	2668	0.94 (0.65 to 1.34)	0.73	13
Self-management	154	1081	232	1035	0.84 (0.53 to 1.35)	0.47	9
Self-testing	335	1676	273	1633	1.23 (1.06 to 1.42)	0.005	4
Thromboembolic events	149	4278	202	4116	0.58 (0.40 to 0.84)	0.004	22*
Self-management	54	2403	106	2237	0.51 (0.37 to 0.69)	<0.0001	15
Self-testing	95	1875	96	1879	0.99 (0.75 to 1.31)	0.95	7
Mortality	197	3323	225	3214	0.83 (0.63 to 1.10)	0.20	13
Self-management	44	1674	68	1619	0.68 (0.46 to 1.01)	0.06	10
Self-testing	153	1649	157	1595	0.97 (0.78 to 1.19)	0.74	3
Time in therapeutic range	NA	2598	NA	2521	WMD 2.82 (0.44 to 5.21)	0.02	11*
Self-management	NA	870	NA	828	WMD 0.47 (−1.40 to 2.34)	0.62	6
Self-testing	NA	1728	NA	1693	WMD 4.44 (1.71 to 7.18)	0.001	5

*For the subgroup meta-analysis according to type of anticoagulant therapy management—, a 4-armed trial, contributed to two studies: one on self-testing and one on self-management.^62^

NA, not applicable; RR, relative risk; WMD, weighted mean difference.

#### Bleeding events

**Figure 3:**
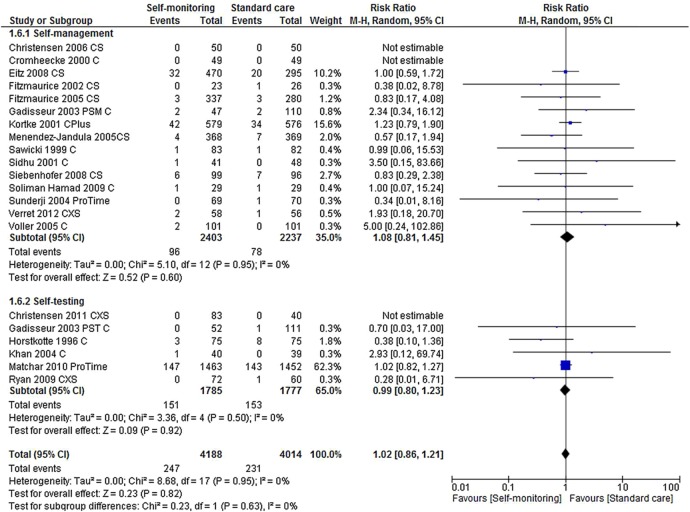
Forest plot of comparison: major bleeding events.

Twenty-one trials reported a total of 1472 bleeding events (major and minor). No statistically significant differences were observed between either self-management or self-testing, and standard monitoring care for major bleeding events (RR 1.08, 95% CI 0.81 to 1.45, p=0.60 and RR 0.99, 95% CI 0.80 to 1.23, p=0.92, respectively) (figure 3 and table 3). Self-testing was associated with a small increased risk of minor bleeding events (RR 1.23, 95% CI 1.06 to 1.42, p=0.005) and all bleeding events (RR 1.15, 95% CI 1.03 to 1.28, p=0.02) while self-management was not (the RR for minor bleeding events was 0.84, 95% CI 0.53 to 1.35, p=0.47 and for all bleeding events 0.94, 95% CI 0.68 to 1.30, p=0.69).

No statistically significant subgroup differences were found for bleeding events by clinical indication for anticoagulant treatment (AHV only, AF only or mixed) or by the setting for standard care (anticoagulant clinics only, physician/GP offices only, or mixed practices).

#### Thromboembolic events

**Figure 4:**
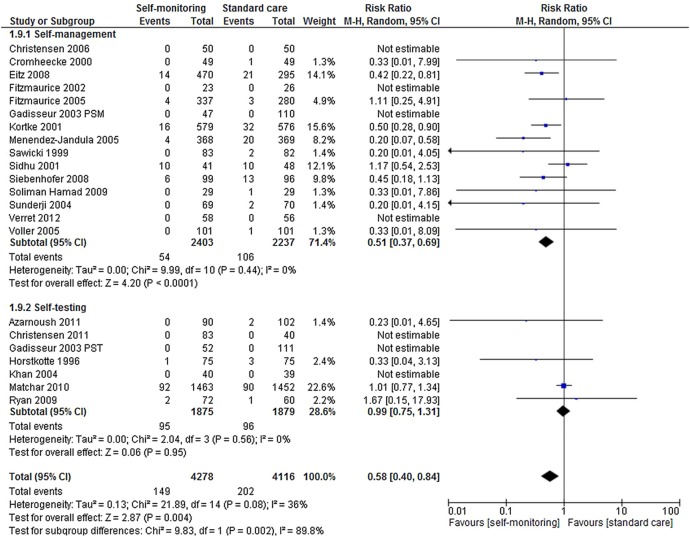
Forest plot of comparison: thromboembolic events.

Twenty-one trials reported a total of 351 thromboembolic events (major and minor) involving 8394 participants.^39–43^ ^45–57^ ^59–62^ Self-monitoring was associated with a statistically significant reduction in the risk of thromboembolic events (RR 0.58, 95% CI 0.40 to 0.84, p=0.004) compared with standard care (figure 4). This reduction was still apparent when the analysis was restricted to major thromboembolic events (RR 0.52, 95% CI 0.34 to 0.80, p=0.003). The reduction in thromboembolic events was observed only in studies of patients carrying out self-management (RR 0.51, 95% CI 0.37 to 0.69, p<0.0001). There was no significant risk reduction among trials of self-testing (RR 0.99, 95% CI 0.75 to 1.31, p=0.56).

The observed reduction in thromboembolic events was similar across clinical indications for anticoagulation: AHV (6 studies, RR 0.56, 95% CI 0.38 to 0.82), AF (2 studies, RR 0.33, 95% CI 0.01 to 8.09) and mixed indications (13 studies, RR 0.57, 95% CI 0.30 to 1.09) (test for subgroup differences: p=0.95). Similarly, there were no significant differences in observed reduction in thromboembolic events among studies which conducted standard care in anticoagulant clinics (10 studies, RR 0.65, 95% CI 0.30 to 1.42), physician/GP offices (6 studies RR 0.45, 95% CI 0.31 to 1.38) or mixed practices (5 studies, RR 0.66, 95% CI 0.31 to 1.38) (test for subgroup differences: p=0.55).

#### Mortality

**Figure 5:**
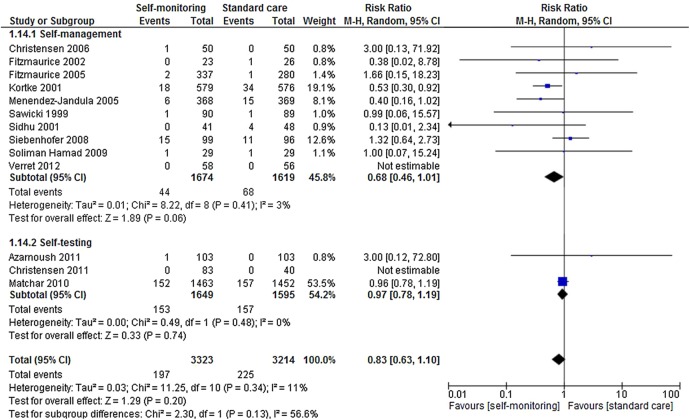
Forest plot of comparison: mortality.

Thirteen trials reported 422 deaths from any cause in a total of 6537 participants.^39^ ^42^ ^43^ ^46^ ^47^ ^49–52^ ^54^ ^56^ ^57^ ^60^ There was no statistically significant difference in all-cause mortality between self-monitoring and standard clinical monitoring (RR 0.83, 95% CI 0.63 to 1.10, p=0.20) (figure 5). Trials of self-management found a reduction in mortality which was close to statistical significance (RR 0.68, 95% CI 0.46 to 1.01, p=0.06), and similar in size and direction to the observed reduction in thromboembolic events. Self-testing had no effect on mortality (RR 0.97 95% CI 0.78 to 1.19, p=0.74).

There was an apparent significant reduction in mortality in trials which restricted entry to patients with AHV (4 trials, RR 0.54, 95% CI 0.32 to 0.92, p=0.02) and no reduction in mortality in trials with mixed clinical indications for anticoagulant therapy (RR 0.95, 95% CI 0.78 to 1.16, p=0.61). As none of the trials reporting mortality specifically excluded patients with AHVs, we could not conclude from the pooled data whether this difference by indication was clinically meaningful.

Deaths directly associated with anticoagulation therapy were reported in five trials.^42^ ^43^ ^47^ ^50^ ^51^ In total, six deaths related to anticoagulation therapy occurred among participants receiving usual monitoring care^42^ ^50^ ^51^ (1 valve thrombosis, 2 myocardial infarctions, 1 retroperitoneal haemorrhage, 1 cerebral haemorrhage, and 1 gastrointestinal bleeding) and seven deaths occurred among participants who self-managed their coagulation status (1 valve thrombosis, 1 pulmonary embolism, 1 massive ischaemic stroke, 2 myocardial infarctions, 1 cerebral haemorrhage, and 1 gastrointestinal bleeding).^43^ ^47^

#### Anticoagulation control: target range

**Figure 6:**
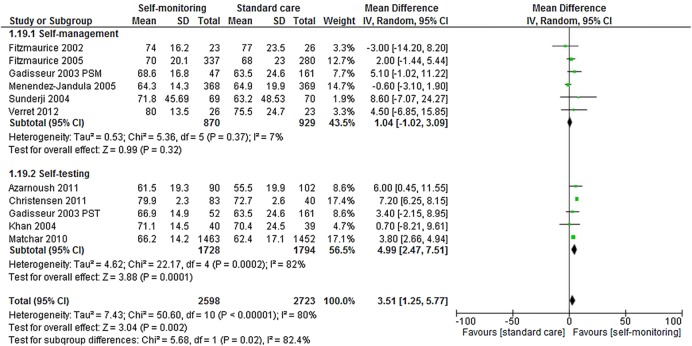
Forest plot of comparison: time in therapeutic range.

Table 4 summarises the results of anticoagulation control reported in the included studies. There was a great variation between trials in the measures used to assess INR time and the values in TTR. In general, INR time and INR values in TTR were reported to be higher among self-monitoring participants compared with those in standard care (table 4). Pooling of INR *values* in TTR across trials proved unfeasible. Eighteen trials^38^ ^39^ ^42^ ^43^ ^47^ ^48^ ^50^ ^51^ ^53–57^ ^59–64^ provided data on INR *time* in TTR and pooling of results was possible for 10 trials that provided suitable data.^42^ ^43^ ^47^ ^53^ ^54^ ^56^ ^57^ ^59^ ^60^ ^62^ No statistically significant differences were observed between self-management and standard care with regard to TTR (p=0.62) (table 3 and figure 6). Nevertheless, a modest but significantly higher proportion of TTR was found for participants who self-tested compared with those who received standard care (WMD 4.44, p=0.001) (table 3 and figure 6).

**Table 4:** INR time and value in therapeutic range

Study ID	INR time in therapeutic range, mean % (SD)			INR value in target range, % of INR values (95% CI)		
	PSM/PST	Control	p Value	PSM/PST	Control	p Value
Azarnoush *et al* 2011^56^	61.5 (19.3)	55.5 (19.9)	0.0343	NR	NR	NR
Bauman *et al* 2010^63^	PSM: 83 (NR) PST: 83.9 (NR)	–	NR	NR	NR	NR
Christensen *et al* 2006^39^	78.7 (69.2–81.0)*	68.9 (59.3–78.2)*	0.14	NR	NR	NR
Christensen 2011^57^	80.2 (2.3)	72.7 (2.6)	<0.001	80.8 (79.3–82.1)	67.2 (64.1–70.2)	<0.001
Cromheecke *et al* 2000^40^	NR	NR	NS	55 (NR)	49 (NR)	0.06
Eitz *et al* 2008^41^	NR	NR		79 (NR)	65 (NR)	<0.001
Fitzmaurice *et al* 2002^42^	74 (16.2)	77 (23.5)	NS	66 (61–71)	72 (65–80)	NS
Fitzmaurice *et al* 2005^43^	70 (20.1)	68 (23.0)	0.18	70 (64.8–74.8)†	72 (66.3 to 77.1)†	NS
Gadisseur *et al* 2003^62^	PSM: 68.6 (16.8) PST: 66.9 (14.9)	67.9 (19.5)	0.33	66.3 (61–71.5)/ 63.9 (59.8–68)‡	61.3 (55–62.4)/58.7‡	0.14
Gardiner *et al* 2006^64^	PSM: 69.9 (23.1) PST: 71.8 (22.1)	–	0.46	NR	NR	NR
Horstkotte *et al* 1996^45^	NR	NR		43.2 (NR)	22.3 (NR)	<0.001
Khan *et al* 2004^59^	71.1 (14.5)	70.4 (24.5)	NS	NR	NR	NR
Kortke *et al* 2001^46^	NR	NR	NR	79.2 (NR)	64.9 (NR)	<0.001
Matchar *et al* 2010^60^	66.2 (14.2)	62.4 (17.1)	<0.001	NR	NR	NR
Menendez-Jandula *et al* 2005^47^	64.3 (14.3)	64.9 (19.9)	0.2	58.6 (SD 14.3)†	55.6 (SD 19.6)†	0.02
Rasmussen *et al* 2012^48^	52 (33–65)§	55 (49–66)	NR	NR	NR	NR
Ryan *et al* 2009^61^	74 (64.6–81)¶	58.6 (45.6–73.1)¶	<0.001	NR	NR	NR
Sawicki 1999^49^	NR	NR	NR	53 (NR)†	43.2 (NR)†	0.22
Sidhu and O'Kane 2001^50^	76.5 (NR)	63.8 (NR)	<0.0001	NR	NR	NR
Siebenhofer *et al* 2008^51^	75.4 (9.4, 85.0)¶	66.5 (47.1, 81.5)¶	<0.001	NR	NR	NR
Soliman Hamad *et al* 2009^52^	NR	NR	NR	72.9 (SD 11)†	53.9 (SD 14)†	0.01
Sunderji *et al* 2004^53^	71.8 (45.69)	63.2 (48.53)	0.14	NR	NR	NR
Verret *et al* 2012^54^	80 (13.5)	75.5 (24.7)	0.79	NR	NR	NR
Völler *et al* 2005^55^	178.8 (126)**	155.9 (118.4)**	NS	67.8 (SD 17.6)	58.5 (SD 19.8)	0.0061

*Median % (95% CI).

†mean % of individual (95% CI).

‡% (95% CI).

§Median % (25–75 centile).

¶Median % (IQR).

**Mean cumulative days (SD).

INR, international normalised ratio; PSM, patient self-management; PST, patient self-testing; NR, not reported; NS, not significant.

The other intermediate outcomes were sparsely reported in the included studies. Two trials reported good patient compliance with self-monitoring (75% and 98%, respectively).^58^ ^59^

### Cost effectiveness

**Figure 7:**
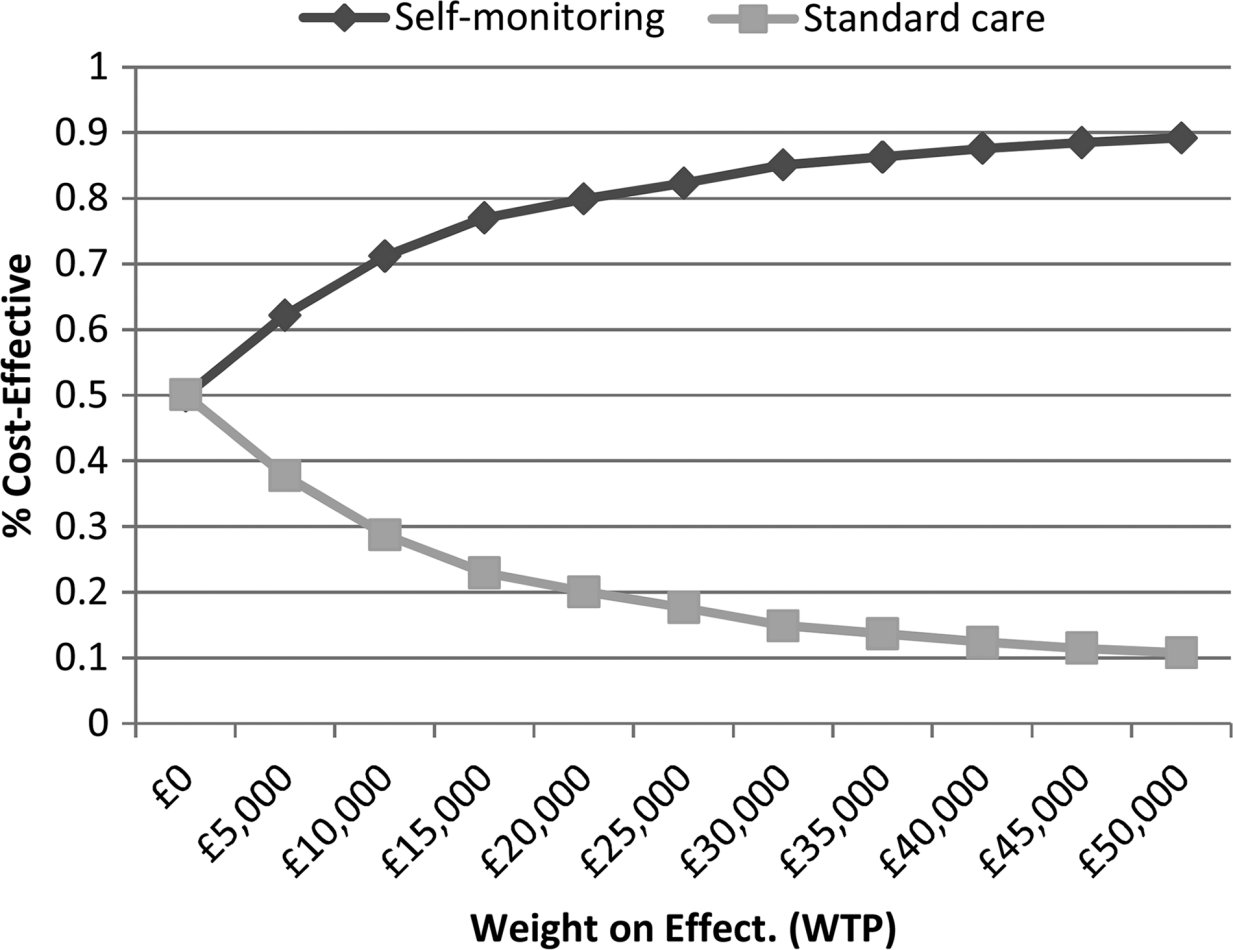
Cost-effectiveness acceptability curves: self-monitoring versus standard care.

Applying the base case assumptions presented in table 1, the results of the cost-effectiveness analyses indicate that over a 10-year period, the introduction of self-monitoring would reduce the proportion of people suffering a thromboembolic event by 2.5%, while slightly increasing the proportion suffering a major haemorrhagic event by 1.2% (table 5). While the predicted monitoring costs are higher with self-monitoring, the total health and social care costs are similar: £7324 for standard care monitoring and £7326 for self-monitoring (table 5). The estimated QALY gain associated with self-monitoring was 0.028. Self-monitoring (50% self-testing, 50% self-management) appears to be cost-effective due to its positive impact on the incidence of thromboembolic events, even though, compared with mixed primary/secondary care, it is likely to increase the INR monitoring costs. Figure 7 shows that self-monitoring as a whole has an approximately 80% chance of being considered cost-effective at a willingness to pay ratio of £20 000 per QALY gained. However, the pooled relative effect estimate for self-testing on thromboembolic events (figure 4) is small and non-significant (RR 0.99), while the effect estimate for self-management is large (RR 0.51) and significant. Thus, within the base case model, self-management alone is highly cost-effective (ie, dominant), while self-testing is not (table 5).

**Table 5:** Mean costs, outcomes and incremental cost-effectiveness over a 10-year time-horizon

Strategy	Mean costs	Cumulative monitoring/ device costs	% with first TE event	% with first major bleed	Mean QALYs	Incremental cost	Incremental QALYs	ICER
Self-monitoring (50% self-management, 50% self-testing) versus standard care								
Standard monitoring	£7324	£1269	14.2	30.2	5.479	–	–	–
Self-monitoring	£7326	£1944	11.7	31.4	5.507	£2	0.028	£71
Base case—100% self-management versus standard care								
Standard monitoring	£7324	£1269	14.2	30.2	5.479	–	–	–
Self-management 100%	£6394	£1717	9.2	32.7	5.535	−£930	0.056	Dominant
Base case—100% self-testing versus standard care								
Standard monitoring	£7324	£1269	14.2	30.2	5.479	–	–	–
Self-testing 100%	£8258	£2171	14.2	30.1	5.479	£934	0	£2 811 298

TE, thromboembolic; QALYs, quality adjusted life years; ICER, incremental cost-effectiveness ratio.

#### Further analysis of uncertainty

In an alternative specification of the model, the overall pooled effect estimates obtained from all self-testing and self-management trials were applied to the self-testing and self-management strategies. Under this scenario, self-monitoring as a whole was found to be cost-saving over standard care (see online supplementary appendix 5 table S3).

Two key parameters underpinning the cost-effectiveness findings are the baseline risk of thromboembolic events, and the relative effect of self-monitoring on these events. The model findings were robust to individual changes in these parameters through feasible ranges. However, when a lower baseline risk of thromboembolic events (1.15%) was combined with the upper 95% confidence limit for the RA associated self-management (RR 0.69), the incremental cost-effectiveness ratio (ICER) for self-monitoring as a whole rose above £30 000 per QALY. The same was found when the lower baseline risk of thromboembolic events was coupled with the upper confidence limit of the pooled RA for self-monitoring (RR 0.89). It should be noted, however, that self-management alone remained cost-saving under the former combined scenario. The cost-effectiveness of self-monitoring improved further when the modelled time horizon was extended to 20 and 30 years, dominating standard primary/secondary care based monitoring. The incremental cost per QALY gained for self-monitoring also remained below £20 000 when higher training failure and discontinuation rates were applied, and when higher self-monitoring testing frequencies were applied (with no change in effects).

Alternative scenarios assessed the potential for self-monitoring to be cost-saving if used to replace clinic-based testing without increasing the frequency of testing (see online supplementary appendix 5 table S4). Under these scenarios it was assumed that there would be no effect on the number of thromboembolic or bleeding events, and a cost-minimisation approach was adopted. This showed that, when holding all other base case parameters constant, self-testing and self-management were more costly than standard primary care monitoring (ie, physician offices and general practices), but less costly than standard secondary care monitoring (ie, specialised anticoagulation clinics).

## Discussion

Our findings suggest that self-monitoring of anticoagulation at home is at least as safe and effective as standard clinic monitoring. Self-management of anticoagulation is associated with reductions in thromboembolic events and possibly, in all-cause mortality. INR time in TTR was reported to be higher in self-monitoring participants compared with standard clinical care. Self-monitoring, and in particular self-management, of anticoagulation status appeared cost-effective at a willingness to pay threshold of £20 000 per QALY gained when pooled estimates of clinical effectiveness were applied to the economic model. The modelled reduction in thromboembolic events was the key driver of cost-effectiveness.

The differences we observed between self-management and self-testing are difficult to explain. Paradoxically, those who self-tested, rather than self-managed, spent more time in TTR (and had more minor bleeding events), but those who self-managed experienced less thromboembolic events. It is possible that people who self-managed their coagulation status take a more active role in managing their therapy or that self-testing leads to more rapid or frequent dose changes. It is also worth noting that the meta-analyses results on self-testing were dominated by the results of the largest trial published so far, the Home International Normalised Ratio Study (THINRS),^60^ which enrolled 2922 people and assessed self-testing versus routine clinical care. This trial had a specialised routine coagulation control and the longest follow-up period (mean 3 years). The high quality of the routine care in the THINRS may exceed current monitoring care for anticoagulation control and could explain the lack of significant differences in major clinical adverse events between self-testing and routine care. When we excluded this trial from the statistical analyses, the risk ratio for thromboembolic events fell from 0.99 to 0.55 among self-testing participants, although the CIs widened (95% CI 0.13 to 2.31).

On the whole, our findings are broadly consistent with those of previously published systematic reviews on self-monitoring using point-of-care devices for the management of anticoagulation therapy, which found that self-monitoring was associated with a significant reduction in the occurrence of thromboembolic events and all-cause-mortality.^14^ ^16^ ^20^ ^27^ ^29^ ^66–69^ Our economic model, in accordance with previous economic evaluations,^29^ ^34^ indicates that self-monitoring is likely to be cost-effective. The findings of our economic model are also broadly in line with those of previous UK-based economic assessments, in that self-monitoring (under base assumptions) will increase the monitoring costs to the NHS. However, our base case differs from that of previous UK evaluations in that the pooled relative effects for self-management and self-testing, compared with standard care, were applied. We observed significant future cost savings and quality of life gains as a consequence of a significant reduction in the incidence of thromboembolic events. This, in turn, translated into more favourable estimates of cost-effectiveness. Further differences between the current analysis and the previous UK-based model include the application of higher standard secondary care monitoring costs, lower self-monitoring device costs (in line with current prices), and higher acute treatment costs for stroke and major bleeding events. Our analyses suggest that the cost-effectiveness of self-monitoring is robust to variations in these parameters when pooled clinical effect estimates are applied to the model.

In more general terms, home monitoring, and especially self-management, of anticoagulation therapy may have a substantial impact on the quality of life of patients and their families. It may reduce the anxiety associated with the fear of deviating from the therapeutic target range and boost confidence in the therapy, increase independence and psychological well-being, and allow for the more efficient organisation of time (eg, travelling, social interactions).

### Limitations

This study has been conducted as per recommended methodological standards and is the most up-to-date evidence synthesis on this topic with the largest number of included RCTs.^20^ ^66^ ^68^

There are, however, potential limitations. The literature searches were performed in 2013 and were not subsequently updated. While the meta-analysis results demonstrated low statistical heterogeneity, which made it statistically reasonable to combine the studies, uncertainties remain that clinical heterogeneity could have contributed to over or underestimate the effects. The included trials varied in terms of clinical indications for anticoagulation therapy, type of control care, reporting structure for the time and/or values in TTR, the mode and structure of the preintervention training and education programme, length of follow-up, and methodological study quality. The majority of the trials included participants with mixed clinical indications for anticoagulation therapy, which made it challenging to extrapolate the results to specific clinical populations. In particular, only limited data were available for people with AF and consequently, no firm conclusions could be drawn in relation to this patient population is. Nevertheless, it likely that self-monitoring may produce similar clinical benefits in people with AF to those achieved in people with artificial heart valves. A great variation between trials was found in the way both INR time and INR values in TTR were measured, which hampered further analyses.

Assuming there is no interaction between the TTR and the relative treatment effect for self-management on thromboembolic events, our modelling suggests that it will remain cost-effective even where TTR is high and the thromboembolic event rate is low. However, it is possible that the quality of standard care may modify the effectiveness of self-monitoring, and in turn, influence its cost-effectiveness. Where patients are already achieving a very high level of INR control, this may limit the potential for self-monitoring to improve TRR and in turn, reduce thromboembolic event. With regard to the economic model, there is still a certain degree of uncertainty surrounding the pooled clinical effectiveness estimates, especially for self-testing. It is worth noting that the perspective of the cost-effectiveness analysis was that of the National Health Service (NHS) and personal social services. Therefore, our modelling fails to capture any wider benefits or cost-savings to patients and their families, such as a reduction in time spent travelling to and waiting in clinics.

Generally, adherence to self-monitoring was reported to be high in the included trials (more than 90%). However, all included trials enrolled highly selected samples of people requiring anticoagulation therapy, and so it was uncertain whether there was strong external validity. To be enrolled in the trials, participants needed to demonstrate adequate cognitive and physical abilities, as well as dexterity and confidence in using the point-of-care device. In some of the included trials^42^ ^43^ ^47^ ^62^ a considerable proportion of eligible participants (up to 50%) ultimately were not considered suitable for inclusion. Despite the enrolment restrictions, results are valid for the patients groups included, which actually represent the population who would be considered for self-monitoring in clinical practices. Six of the trials were conducted in the UK and we could not find any evidence that the UK trial patient cohorts were fundamentally different from those of the rest of the included studies.

Whilst new non-vitamin K antagonist oral anticoagulants were beyond the scope of this assessment, these offer an alternative option for many people with AF who are currently on warfarin. However, these are not suitable for all people who need anticoagulation therapy. Furthermore, due to the potential risk of bleeding, it is unlikely that people receiving warfarin who have stable INR may switch to the NOACs. Therefore, there are still many people who receive warfarin rather than the NOACs for whom self-monitoring is still of clinical relevance.

## Conclusions

Self-monitoring, and in particular self-management, is a safe and cost-effective option for people requiring long-term vitamin K antagonist therapy. Further research assessing the longer-term outcomes of self-management versus standard monitoring care as well as the comparative effectiveness of various point-of-care coagulometers would be useful. The technology related to these devices is constantly changing and future research needs to target larger cohorts of people with different clinical indications requiring long-term anticoagulation therapy. It is worth acknowledging that the modern point-of-care coagulometers are likely to have advanced both in their ease of use and cost, which, in theory, could modify the possible candidates for these devices as well as the magnitude of any economic evaluation.

## References

[1] DeWilde S, Carey IM, Emmas C Trends in the prevalence of diagnosed atrial fibrillation, its treatment with anticoagulation and predictors of such treatment in UK primary care. Heart 2006;92:1064–70. 10.1136/hrt.2005.069492 16387813 PMC1861124

[2] Camm AJ, Kirchhof P, Lip GY, European Heart Rhythm Association, European Association for Cardio-Thoracic Surgery. Guidelines for the management of atrial fibrillation: the Task Force for the Management of Atrial Fibrillation of the European Society of Cardiology (ESC). Eur Heart J 2010;31:2369–429. 10.1093/eurheartj/ehq278 20802247

[3] Cowan C, Healicon R, Robson I The use of anticoagulants in the management of atrial fibrillation among general practices in England. Heart 2013;99:1166–72. 10.1136/heartjnl-2012-303472 23393083 PMC3717828

[4] Heart valve disease [website on the Internet]. London: British Heart Foundation, 2013 http://www.bhf.org.uk/heart-health/conditions/heart-valve-disease.aspx (accessed Apr 2015).

[5] Aortic stenosis [website on the Internet]. Leeds: Patient UK, 2013 http://www.patient.co.uk/health/aortic-stenosis (accessed Apr 2015).

[6] UK Heart Valve Registry [archived webpage on the Internet]. London: NHS Health and Social Care Information Centre, 2010 http://docdat.ic.nhs.uk/DatabaseList.aspx (accessed Apr 2015).

[7] Cayley J Self-monitoring and self-management of anticoagulation therapy. Am Fam Physician 2011;84:266–8. 21842772

[8] Anticoagulation therapy service commissioning guide [document on the Internet]. London: National Insititute for Health and Care Excellence, 2010 URL:http://www.nice.org.uk/usingguidance/commissioningguides/anticoagulationtherapyservice/anticoagulationtherapyservice.jsp (accessed Apr 2015).

[9] Atrial fibrillation [website on the Internet]. London: British Heart Foundation, 2013 http://www.bhf.org.uk/heart-health/conditions/atrial-fibrillation.aspx (accessed Apr 2015).

[10] SIGN 129 Antithrombotics: indications and management [document on the Internet]. Edinburgh: Health Improvement Scotland, 2013 http://www.sign.ac.uk/pdf/SIGN129.pdf (accessed April 2015).

[11] Dabigatran etexilate for the prevention of stroke and systemic embolism in atrial fibrillation. NICE Guidance TA249 [document on the Internet]. London: National Insititute for Health and Care Excellence, 2012 http://guidance.nice.org.uk/TA249 (accessed Apr 2015). 10.1136/heartjnl-2012-302101 22698856

[12] Fuster V, Ryden LE, Cannom DS ACC/AHA/ESC 2006 guidelines for the management of patients with atrial fibrillation: full text: a report of the American College of Cardiology/American Heart Association Task Force on practice guidelines and the European Society of Cardiology Committee for Practice Guidelines (Writing Committee to Revise the 2001 guidelines for the management of patients with atrial fibrillation) developed in collaboration with the European Heart Rhythm Association and the Heart Rhythm Society. Europace 2006;8:651–745. 10.1093/europace/eul097 16987906

[13] You JJ, Singer DE, Howard PA Antithrombotic therapy for atrial fibrillation: Antithrombotic Therapy and Prevention of Thrombosis, 9th ed: American College of Chest Physicians Evidence-Based Clinical Practice Guidelines. Chest 2012;141 (2 Suppl):e531S–75S. 22315271 10.1378/chest.11-2304 PMC3278056

[14] Connock M, Stevens C, Fry-Smith A Clinical effectiveness and cost-effectiveness of different models of managing long-term oral anticoagulation therapy: a systematic review and economic modelling. Health Technol Assess 2007;11:iii–iv, ix–66. 10.3310/hta11380 17903392

[15] Ansell J, Jacobson A, Levy J International Self-Monitoring Association for Oral Anticoagulation. Guidelines for implementation of patient self-testing and patient self-management of oral anticoagulation. International consensus guidelines prepared by International Self-Monitoring Association for Oral Anticoagulation. Int J Cardiol 2005;99:37–45. 10.1016/j.ijcard.2003.11.008 15721497

[16] Point-of-Care Testing: A Review of Systematic Reviews on Testing Accuracy and Cost-Effectiveness [document on the Internet]. Ottawa: Canadian Agency for Drugs and Technologies in Health, 2012 http://www.cadth.ca/media/pdf/htis/april-2012/RC0345%20Point%20of%20Care%20Testing%20Final.pdf (accessed Apr 2015).

[17] Christensen TD, Larsen TB Precision and accuracy of point-of-care testing coagulometers used for self-testing and self-management of oral anticoagulation therapy. J Thromb Haemost 2012;10:251–60. 10.1111/j.1538-7836.2011.04568.x 22118602

[18] Tamayo AE, Vergara-Mitxeltorena I, Uranga Saez del BE Oral anticoagulation and self-management: Analysis of the factors that determine the feasibility of using self-testing and self-management in primary care. BMC Cardiovasc Disorder 2013;13:59 10.1186/1471-2261-13-59 PMC3765739 23968316

[19] Jones S, Monagle P, Manias E Quality of life assessment in children commencing home INR self-testing. Thromb Res 2013;132:37–43. 10.1016/j.thromres.2013.05.011 23726963

[20] Garcia-Alamino JM, Ward AM, Alonso-Coello P Self-monitoring and self-management of oral anticoagulation. Cochrane Database Syst Rev 2010;(4):CD003839. doi:10.1002/14651858.CD003839.pub2. 20393937 10.1002/14651858.CD003839.pub2

[21] Systematic reviews: CRD's guidance for undertaking systematic reviews in health care [document on the Internet]. University of York: Centre for Reviews and Dissemination, 2009 http://www.york.ac.uk/inst/crd/SysRev/!SSL!/WebHelp/SysRev3.htm (accessed April 2015).

[22] Higgins JP, Green S Cochrane Handbook for Systematic Reviews of Interventions Version 5.1.0 [document on the Internet]. The Cochrane Collaboration, 2011 http://www.cochrane-handbook.org/ (accessed April 2015).

[23] National Institute for Health and Care Excellence. Guide to the methods of technology appraisal [document on the Internet]. London: National Institute for Health and Care Excellence, 2013 http://publications.nice.org.uk/guide-to-the-methods-of-technology-appraisal-2013-pmg9 (accessed Apr 2015). 27905712

[24] Moher D, Liberati A, Tetzlaff J PRISMA Group. Preferred reporting items for systematic reviews and meta-analyses: the PRISMA statement. J Clin Epidemiol 2009;62:1006–12. 10.1016/j.jclinepi.2009.06.005 19631508

[25] Sonnenberg FA, Beck JR Markov models in medical decision making: a practical guide. Med Decis Making 1993;13:322–38. 10.1177/0272989X9301300409 8246705

[26] Beck JR, Pauker SG The Markov process in medical prognosis. Med Decis Making 1983;3:419–58. 10.1177/0272989X8300300403 6668990

[27] Point-of-Care International Normalized Ratio (INR) Monitoring Devices for Patients on Long-term Oral Anticoagulation Therapy. Ontario Health Asssessment Series 9 (12) [document on the Internet]. Ontario: Medical Advisory Secretariat Ministry of Health and Long-Term Care, 2009 http://www.health.gov.on.ca/english/providers/program/mas/tech/reviews/pdf/rev_poc_20090928.pdf (accessed Apr 2015). PMC3377545 23074516

[28] Brown A, Wells P, Jaffey J Point-of-Care Monitoring Devices for Long-Term Oral Anticoagulation Therapy: Clinical and Cost Effectiveness. Technology Report no 72 [document on the Internet]. Ottawa: Canadian Agency for Drugs and Technologies in Health, 2007 http://www.cadth.ca/media/pdf/H0299_anticoagulation-therapy_tr_e.pdf (accessed Apr 2015).

[29] Gailly J, Gerkens S, van den Bruel A Use of point-of-care devices in patients with oral anticoagulation: a Health Technology Assessment. KCE Report 117C [document on the Internet]. Brussels: Belgian Health Care Knowledge Centre, 2009 https://kce.fgov.be/publication/report/use-of-point-of-care-devices-in-patients-with-oral-anticoagulation-a-health-techn (accessed Apr 2015).

[30] Lafata JE, Martin SA, Kaatz S The cost-effectiveness of different management strategies for patients on chronic warfarin therapy. J Gen Intern Med 2000;15:31–7. 10.1046/j.1525-1497.2000.01239.x 10632831 PMC1495325

[31] Muller E, Bergemann R, GELIA Study Group. Economic analysis of bleeding and thromboembolic sequelae after heart valve replacement (GELIA 7). Eur Heart J Suppl 2001;3 (Suppl Q):65–9. 10.1016/S1520-765X(01)90046-X

[32] Regier DA, Sunderji R, Lynd LD Cost-effectiveness of self-managed versus physician-managed oral anticoagulation therapy. CMAJ 2006;174:1847–52. 10.1503/cmaj.051104 16785459 PMC1475919

[33] Sola-Morales O Portable coagulometers: revision of the scientific evidence and economic assessment of their use in self-control of oral anticoagulant treatment. Barcelona: Catalan Agency for Health Technology Assessment and Research (CAHTA), 2003.

[34] Taborski U, Wittstamm FJ, Bernardo A Cost-effectiveness of self-managed anticoagulant therapy in Germany. Semin Thromb Hemost 1999;25:103–7. 10.1055/s-2007-996432 10327229

[35] Rivaroxaban for the prevention of stroke and systemic embolism in people with atrial fibrillation NICE Guidance TA256 [document on the Internet]. London: National Insititute for Health and Care Excellence, 2012 http://guidance.nice.org.uk/TA256 (accessed Apr 2015).

[36] NICE Diagnostic Assessment Programme manual [document on the Internet]. London: National Institute for Health and Care Excellence, 2011 http://www.nice.org.uk/media/A0B/97/DAPManualFINAL.pdf (accessed April 2015). 27466648

[37] NHS reference costs 2011–12 [website on the Internet]. UK Department of Health, 2012 https://www.gov.uk/government/publications/nhs-reference-costs-financial-year-2011-to-2012 (accessed Apr 2015).

[38] Curtis L Unit Costs of Health and Social Care 2013 [document on the Internet]. Canterbury: Personal Social Services Research Unit, 2012 http://www.pssru.ac.uk/project-pages/unit-costs/2012/ (accessed Apr 2015).

[39] Christensen TD, Maegaard M, Sorensen HT Self-management versus conventional management of oral anticoagulant therapy: a randomized, controlled trial. Eur J Intern Med 2006;17:260–6. 10.1016/j.ejim.2005.11.021 16762775

[40] Cromheecke ME, Levi M, Colly LP Oral anticoagulation self-management and management by a specialist anticoagulation clinic: a randomised cross-over comparison. Lancet 2000;356:97–102. 10.1016/S0140-6736(00)02470-3 10963245

[41] Eitz T, Schenk S, Fritzsche D International normalized ratio self-management lowers the risk of thromboembolic events after prosthetic heart valve replacement. Ann Thorac Surg 2008;85: 949–54. 10.1016/j.athoracsur.2007.08.071 18291177

[42] Fitzmaurice DA, Murray ET, Gee KM A randomised controlled trial of patient self management of oral anticoagulation treatment compared with primary care management. J Clin Pathol 2002;55:845–9. 10.1136/jcp.55.11.845 12401823 PMC1769803

[43] Fitzmaurice DA, Murray ET, McCahon D Self management of oral anticoagulation: randomised trial. BMJ 2005;331:1057 10.1136/bmj.38618.580903.AE 16216821 PMC1283185

[44] Hemkens LG, Hilden KM, Hartschen S A randomized trial comparing INR monitoring devices in patients with anticoagulation self-management: evaluation of a novel error-grid approach. J Thromb Thrombol 2008;26:22–30. 10.1007/s11239-007-0070-4 17965836

[45] Horstkotte D, Piper C, Wiemer M Improvement of prognosis by home prothrombin estimation In patients with life-long anticoagulant therapy. Eur Heart J 1996;17:230.8732376

[46] Kortke H, Minami K, Breymann T INR self-management after mechanical heart valve replacement: ESCAT (Early Self-Controlled Anticoagulation Trial). Z Kardiol 2001;90 (Suppl 6):VI/118–24. 10.1007/s003920170019 24445799

[47] Menéndez JB, Souto JC, Oliver A Comparing self-management of oral anticoagulant therapy with clinic management: a randomized trial. Ann Intern Med 2005;142:1–10. 10.7326/0003-4819-142-1-200501040-00006 15630104

[48] Rasmussen RS, Corell P, Madsen P Effects of computer-assisted oral anticoagulant therapy. Thromb J 2012;10:17 10.1186/1477-9560-10-17 22935243 PMC3502261

[49] Sawicki PT A structured teaching and self-management program for patients receiving oral anticoagulation: a randomized controlled trial. Working Group for the Study of Patient Self-Management of Oral Anticoagulation. JAMA 1999;281:145–50. 10.1001/jama.281.2.145 9917117

[50] Sidhu P, O'Kane HO Self-managed anticoagulation: results from a two-year prospective randomized trial with heart valve patients. Ann Thorac Surg 2001;72:1523–7. 10.1016/S0003-4975(01)03049-1 11722037

[51] Siebenhofer A, Rakovac I, Kleespies C, SPOG 6. Self-management of oral anticoagulation reduces major outcomes in the elderly. A randomized controlled trial. Thromb Haemost 2008;100:1089–98. 19132235

[52] Soliman H, van E, van A Self-management program improves anticoagulation control and quality of life: a prospective randomized study. Eur J Cardiothorac Surg 2009;35:265–9. 10.1016/j.ejcts.2008.10.020 19041254

[53] Sunderji R, Gin K, Shalansky K A randomized trial of patient self-managed versus physician-managed oral anticoagulation. Can J Cardiol 2004;20:1117–23. 15457308

[54] Verret L, Couturier J, Rozon A Impact of a pharmacist-led warfarin self-management program on quality of life and anticoagulation control: a randomized trial. Pharmacotherapy 2012;32:871–9. 10.1002/j.1875-9114.2012.01116 23033226

[55] Völler H, Glatz J, Taborski U Self-management of oral anticoagulation in nonvalvular atrial fibrillation (SMAAF study). Zeitschrift für Kardiologie 2005;94:182–6. 10.1007/s00392-005-0199-0 15747040

[56] Azarnoush K, Camilleri L, Aublet-Cuvelier B Results of the first randomized French study evaluating self-testing of the International Normalized Ratio. J Heart Valve Dis 2011;20:518–25. 22066355

[57] Christensen TD Self-management of oral anticoagulation therapy—methodological and clinical aspects. Dan Med Bull 2011;58:B4284. 21535992

[58] Gardiner C, Williams K, Mackie IJ Patient self-testing is a reliable and acceptable alternative to laboratory INR monitoring. Br J Haematol 2005;128:242–7. 10.1111/j.1365-2141.2004.05300.x 15638860

[59] Khan TI, Kamali F, Kesteven P The value of education and self-monitoring in the management of warfarin therapy in older patients with unstable control of anticoagulation. Br J Haematol 2004;126:557–64. 10.1111/j.1365-2141.2004.05074.x 15287950

[60] Matchar DB, Jacobson A, Dolor R Effect of home testing of international normalized ratio on clinical events. N Engl J Med 2010;363:1608–20. 10.1056/NEJMoa1002617 20961244

[61] Ryan F, Byrne S, O'Shea S Randomized controlled trial of supervised patient self-testing of warfarin therapy using an internet-based expert system. J Thromb Haemost 2009;7:1284–90. 10.1111/j.1538-7836.2009.03497.x 19496921

[62] Gadisseur AP, Breukink-Engbers WG, Meer FJ Comparison of the quality of oral anticoagulant therapy through patient self-management and management by specialized anticoagulation clinics in the Netherlands: a randomized clinical trial. Arch Intern Med 2003;163:2639–46. 10.1001/archinte.163.21.2639 14638565

[63] Bauman ME, Black K, Bauman ML EMPoWarMENT: Edmonton pediatric warfarin self-management pilot study in children with primarily cardiac disease. Thromb Res 2010;126:e110–15. 10.1016/j.thromres.2010.05.024 20584541

[64] Gardiner C, Williams K, Longair I A randomised control trial of patient self-management of oral anticoagulation compared with patient self-testing. Br J Haematol 2006;132:598–603. 10.1111/j.1365-2141.2005.05899.x 16445833

[65] Siebenhofer A, Rakovac I, Kleespies C Self-management of oral anticoagulation in the elderly: rationale, design, baselines and oral anticoagulation control after one year of follow-up. A randomized controlled trial. Thromb Haemost 2007;97:408–16. 17334508

[66] Bloomfield HE, Krause A, Greer N Meta-analysis: effect of patient self-testing and self-management of long-term anticoagulation on major clinical outcomes. Ann Intern Med 2011;154:472–82. 10.7326/0003-4819-154-7-201104050-00005 21464349

[67] Heneghan C, Alonso-Coello P, Garcia-Alamino JM Self-monitoring of oral anticoagulation: a systematic review and meta-analysis. Lancet 2006;367:404–11. 10.1016/S0140-6736(06)68139-7 16458764

[68] Heneghan C, Ward A, Perera R Self-monitoring of oral anticoagulation: systematic review and meta-analysis of individual patient data. Lancet 2012;379:322–34. 10.1016/S0140-6736(11)61294-4 22137798

[69] Wells PS, Brown A, Jaffey J Safety and effectiveness of point-of-care monitoringdevices in patients on oral anticoagulant therapy: a meta-analysis. Open Medicine 2007;1:E131–46. 21673942 PMC3113217

[70] Bayer PLC Single Technology Appraisal (STA) of Rivaroxaban (Xarelto®) [document on the Internet]. London: National Institute for Health and Care Excellence, 2011 http://guidance.nice.org.uk/TAG/274/Consultation/EvaluationReport/ManufacturerSubmissions/Bayer/pdf/English (accessed Apr 2015).

[71] Jowett S, Bryan S, Murray E Patient self-management of anticoagulation therapy: a trial-based cost-effectiveness analysis. Br J Haematol 2006;134:632–9. 10.1111/j.1365-2141.2006.06243.x 16938120

